# Steps Toward Engagement Integrity: Learning From Participatory Visual Methods in Marginalized South African Communities

**DOI:** 10.3389/fpubh.2022.794905

**Published:** 2022-06-27

**Authors:** Gillian F. Black, Pam Sykes

**Affiliations:** ^1^Sustainable Livelihoods Foundation, Cape Town, South Africa; ^2^Independent Researcher, Vancouver, BC, Canada

**Keywords:** community engagement and involvement, water crisis, two-way communication, ethics, participatory visual methods, hand mapping, body mapping, engagement integrity

## Abstract

Community engagement and involvement have been increasingly recognized as an ethical and valuable component of health science research over the past two decades. Progress has been accompanied by emerging standards that emphasize participation, two-way communication, inclusion, empowerment, and ownership. Although these are important and noble benchmarks, they can represent a challenge for research conducted in marginalized contexts. This community case study reports on the methods, outcomes, constraints and learning from an NGO-led community engagement project called Bucket Loads of Health, implemented in the Western Cape province of South Africa. The independent project team used multiple participatory visual methods to foster two-way communication between members of two disenfranchised communities, Enkanini and Delft, and a group of water microbiologists at Stellenbosch University who were conducting research in Enkanini. The project was carried out during the 2018 Western Cape water crisis, under the growing threat of “Day Zero”. The resulting visual outputs illustrated the negative impacts of water shortage on health and wellbeing in these community settings and showcased scientific endeavors seeking to address them. Engagement included knowledge exchange combining body maps, role play performances and films created by the community members, with hand maps, posters and presentations produced by the scientists. Whereas these engagement tools enabled reciprocal listening between all groups, their ability to respond to the issues raised was hindered by constraints in resources and capacity beyond their control. An additional core objective of the project was to bring the impacts of water shortage in participating communities, and the work of the research team, to the attention of local government. The case study demonstrates the challenges that politically ambitious community engagement faces in being acknowledged by government representatives. We further the argument that research institutions and funders need to match professed commitments to engagement with training and resources to support researchers and community members in responding to the needs and aspirations surfaced through engagement processes. We introduce the concept of engagement integrity to capture the gap between recommended standards of community engagement and what is realistically achievable in projects that are constrained by funding, time, and political interest.

## Introduction

Fieldwork for health-related scientific exploration is largely done in settings where the health challenge under investigation has a direct impact. The intention is to improve the health and wellbeing of people experiencing that impact, plus others who reside in similar settings and face the same challenges. Historically, health science research has been designed and implemented by scientists, with minimal engagement or involvement of those who live in the communities where their fieldwork is conducted, other than enrolling them as research participants. Over the past two decades, community engagement and involvement (CEI) has been increasingly recognized as an ethical and valuable component of health research (1, 2). Research approval by funders and institutional review boards is becoming more dependent on CEI being embedded into the proposed activities, and funding for CEI in global health research is more readily available.

UNICEF and others have proposed that core standards of engagement should include community participation, empowerment and ownership, inclusion, two-way communication, adaptability and localization, and should also build on local capacity (3, 4). These are crucial ethical standards, and all of them need to be integrated into CEI initiatives to achieve engagement integrity. By “Engagement Integrity” we mean a situation in which the good intentions of CEI are achieved to the extent that community members end an engagement process in a position of greater knowledge, capacity, power, and inclusion than they started. For example, according to UNICEF's core standards, two-way communication calls for “communities to be able to provide feedback as an indicator of project success” and adaptability and localization require “CEI approaches to be flexible and responsive to local populations' needs, conditions, and concerns”. These are ambitious requirements for a field that is still emerging within global health research practice and, as this paper demonstrates, achieving them is not straightforward. UNICEF's core standards (3) provided a framework for us to reflect on the possibilities and challenges of community engagement through the South African case study reported here.

Good two-way communication requires those who are involved to participate in a process of listening and responding that is open, balanced and reciprocal (5). This suggests that research engagement should aim to cultivate a genuine and equal exchange of knowledge and perspectives between multiple stakeholder groups. However, enabling this type of communication within a research project can pose a significant challenge, especially when the research is being done in marginalized settings, the project is not resourced with a dedicated CEI team, and the research group has not received training in CEI.

Participatory visual methods (PVM) provide ways to generate materials that foster good two-way communication and can strengthen co-learning in research engagement initiatives (6). In a PVM process, focus group members create visual materials and/or performances to illustrate their lived experiences and convey their individual and collective perspectives on a situation. These materials then provide platforms for engagement across which opinions, ideas, needs and aspirations can be exchanged, discussed and debated.

### The Rationale for the Innovation

This community case study reports on the methods, outcomes, constraints and learning from a project called Bucket Loads of Health (BLH) which aimed to promote community engagement in water microbiology research in the Western Cape province of South Africa. BLH was implemented in 2018 while the province was experiencing its worst drought in over 100 years, raising the prospect of a “Day Zero” when piped water supplies would be shut off (7). Extreme water restrictions resulted in the widespread use of greywater and untreated rainwater for watering gardens, washing clothes and dishes, and flushing toilets. This raised several public health concerns which were the catalyst for the BLH project.

The BLH project was conceptualized and led by a representative of the Sustainable Livelihoods Foundation (SLF)^1^ with prior experience in using PVM and facilitating community engagement in health science research (8–10). With the support of a Wellcome International Engagement Award, SLF invited a team of microbiologists from the Water Resource Laboratory (WRL) at Stellenbosch University (SUN) to be the research partners in the BLH project. Two consultants with experience in community engagement and participatory methods (visual and musical) supported SLF with the design and facilitation of the BLH project activities (11–13). SLF and supporting consultants are hereafter named as the engagement team.

A core goal of the WRL is to generate alternative, sustainable and safe sources of water through the *in-situ* solar pasteurization and solar disinfection of rainwater harvested from the roofs of shacks in informal settlements (14). At the time of the BLH project, the microbiologists were doing research in the informal settlement of Enkanini near the university. They did not have pre-existing engagement support and had limited resources to interact with community members. The BLH project enabled the microbiology team to creatively engage with a focus group of Enkanini residents. BLH also allowed the scientists to engage with residents of Delft, a large township in Cape Town. The microbiology team were not conducting research in Delft at the time, but residents of the township were heavily impacted by the 2018 water restrictions and had existing relationships with SLF making this an appropriate location for additional engagement. SLF and the WRL partnered for the first time in the BLH project.

The engagement team took a PVM approach to facilitate two-way communication between the community focus groups and the water scientists. This article describes how multiple visual methods were layered into the project and the ways in which the outputs provided effective interfaces for knowledge exchange and co-learning. We also examine the limitations of the project and propose how our learning can contribute to progress in the field of CEI, shedding light on what is needed to advance toward engagement integrity.

## Context

Enkanini is located on the outskirts of Stellenbosch, a prosperous university town. Enkanini—the name is isiXhosa for “taken by force”—was created in 2005 when a group of people from the neighboring township of Kayamandi invaded and built shacks on vacant municipal land. It is estimated that as of 2013, Enkanini was home to between 8,000 and 10,000 people, with current population figures unknown (15).

As an unplanned informal settlement, Enkanini has no sewerage or wastewater infrastructure, and stormwater drainage is absent. Individual shacks have no formal connection to municipal water or electricity services, although there are many illegal connections. By 2013, the settlement had 32 communal taps and 80 waterborne toilets in combined water and sanitation blocks (15). Residents live in a state of perpetual water scarcity, punctuated by floods after heavy rainfall.

Delft is a much larger settlement built between 1996 and 2000 under South Africa's post-apartheid Reconstruction and Development Programme (16). The population of Delft is ~ 152,000; about 47% of residents speak Afrikaans as their first language, 38% speak isiXhosa and 9% speak English (17). Although Delft is moderately well served by formal municipal infrastructure compared to Enkanini, it is socially fractured and subject to high levels of violent crime (8, 18).

Paradoxically, Delft's integration with formal municipal systems increased pressure on residents during the 2018 water crisis. The City of Cape Town municipality (CoCT) implemented extreme water-saving measures including a ration of 50 L per person per days, enforced in some cases by automatic shutoffs at the level of household water meters; reduced flow to entire neighborhoods; and increased tariffs for higher usage. This made a minimal difference in informal settlements, where the need to carry water from communal standpipes had in many cases already limited residents to around 50 L per days (19).

## Mobilization of Focus Groups

In Enkanini, a community leader who lived in the settlement and had previously worked as a community-based researcher for the WRL was chosen by the microbiology team to mobilize the group of Enkanini participants for the BLH project. In Delft, participant mobilization was done by a community leader who lived in the township and had taken part as a research participant in earlier research projects run by SLF. These community leaders were both given an outline of the goals, methods and timeline of the BLH project and asked to identify up to 15 males and females in their respective communities who were over the age of 18, available and interested to take part according to the project outline provided.

The Enkanini focus group comprised 12 residents, all first-language isiXhosa speakers originally from the Eastern Cape. There were 8 women ranging in age from 18 to 44 and 4 men ranging in age from 23 to 35. The length of time spent living in Enkanini differed among the participants. However, they had all lived in the settlement for over 12 months, having moved there from other informal settlement contexts. The maximum level of formal education among the group was matriculation from high school. None of the Enkanini participants were in sustained employment at the time of the project; their incomes came from government grants and occasional piece work, for example as domestic workers or unskilled laborers. Three participants had previously worked as co-researchers on Stellenbosch University projects conducted in their community, and two had worked with the microbiology team to facilitate stakeholder engagement.

The Delft focus group comprised 15 residents of the township, including three isiXhosa participants originally from the Eastern Cape and 12 participants whose first language was Afrikaans. There were 10 women ranging in age from 23 to 64 and 5 men ranging in age from 27 to 58. Amongst the participant group, the length of time spent living in Delft ranged from 10 to 20 years. As with the Enkanini participants, the maximum level of formal education among the Delft group was matriculation from high school. Some members of the group had part time employment through schools, non-governmental or community-based organizations, though the majority were unemployed and depended on government grants for income. Two members of the group had an existing relationship with SLF through involvement in previous research projects.

The research partners included the group leader, Professor Wesaal Khan, and four members of her research laboratory, including one post-doctoral fellow, two PhD students and a Masters student.

The participants in both community settings, and the microbiology team, committed to the project throughout its one-year duration. The Delft and Enkanini groups functioned independently and did not meet.

## Key Programmatic Elements

The key programmatic elements of the BLH project incorporated three main phases: workshops, knowledge exchange days and public exhibition events. The key content of these phases is outlined below. Workshop and meeting agendas were flexible and provided opportunities for reflection and adjustment, with the intention that participants should have substantial control of the project outcomes. The engagement team was conscious of several barriers to effective two-way communication, including differences in race, multiple first languages, and vast differentials in income, formal education, expertise, experience in public speaking, and power. All activities and events were designed with these differences in mind and were facilitated by the engagement team. In Delft, the existing relationships between SLF and community organizations also helped to mitigate these barriers.

## Phase 1: Workshops

Separate workshops were held with the focus groups from the two participating communities and the microbiology team.

### Community Focus Groups

#### Inception Workshops

In both Enkanini and Delft, the engagement process began with a one-day inception workshop. This allowed for introductions, a more-in-depth explanation of the goals, methods, and timeline of the project by the engagement team and an opportunity for attendees to ask questions. The inception workshops included a session to review the hopes, fears, and expectations of the potential participants with the intention of managing expectations around the possibilities and limitations of the BLH project. Expectations of project outcomes were revisited and discussed throughout the different phases and activities of the engagement process. Those who wished to participate in the project were asked to give their written consent. Most of the community members who attended the inception workshops in both settings consented to participate in the entire project.

In Enkanini, participants expressed their interest in BLH as wanting to know more about the water research that they could see taking place in their settlement and to understand any direct benefits for the wider community from this research. In Delft, participants said that they were interested to take part in the project because they wanted to be informed about research that was being done to address a household problem that was severely affecting them and their community. In both settings the focus group members expressed enthusiasm for sharing their lived experience of water shortage with researchers and other stakeholders as they had not previously been given an opportunity to do this.

#### Creative Workshops

Due to poor standards of education and limited employment opportunities for those living in marginalized settings, it was expected that participants from Enkanini and Delft might face difficulty with reading and writing. Differences in first language between the community participants, the microbiologists and the engagement team also brought about a barrier to effective engagement. As reported elsewhere, (20) visual forms of communication can aid challenges of literacy and language. They can also help to balance the dynamics of power and knowledge that are likely to exist when “non-expert” community members are brought into discussion with professionals. Hence a PVM approach with optional writing activities was followed in the BLH project. The creative workshops took place over five full consecutive days in community halls in Stellenbosch and Delft. Activities included the creation of sound, images, movement, performance and video, as well as opportunities for reflection and feedback. Storytelling and story sharing was woven throughout, into almost every activity. Being familiar with informal settlement and township contexts, we considered it unlikely that participants' schooling had included many opportunities for image-making, so paints, pastels and markers were introduced gradually to enable participants to build confidence.

#### Community Mapping

Early in each workshop, the whole group worked together to create a color-coded water typology, describing the forms in which they encountered water in their daily lives. This included clean tap water, stormwater, sewerage, wastewater from washing, rivers, and streams, puddles and industrial runoff. Small groups then created hand-drawn community maps, highlighting places of importance to them and places where they encountered water in its various forms. This exercise surfaced stories and experiences that were later incorporated into body maps.

#### Body Mapping

Body mapping was first described as a research method in a comparison of women's identity and the concept of the reproductive system in rural Jamaica and the UK (21). In 2002, Cornwall reported on body mapping as an exercise to build connections between different types of experience and knowledge, including biomedical messages, when exploring sexual and reproductive health with women in Zimbabwe (22). MacGregor has discussed the use of body mapping as a tool for “context sensitive” science education among people living with HIV/AIDS in South Africa (23). Body mapping has also been used as a qualitative method in the fields of social justice, knowledge translation and therapeutic benefit (24).

**Figure 1:**
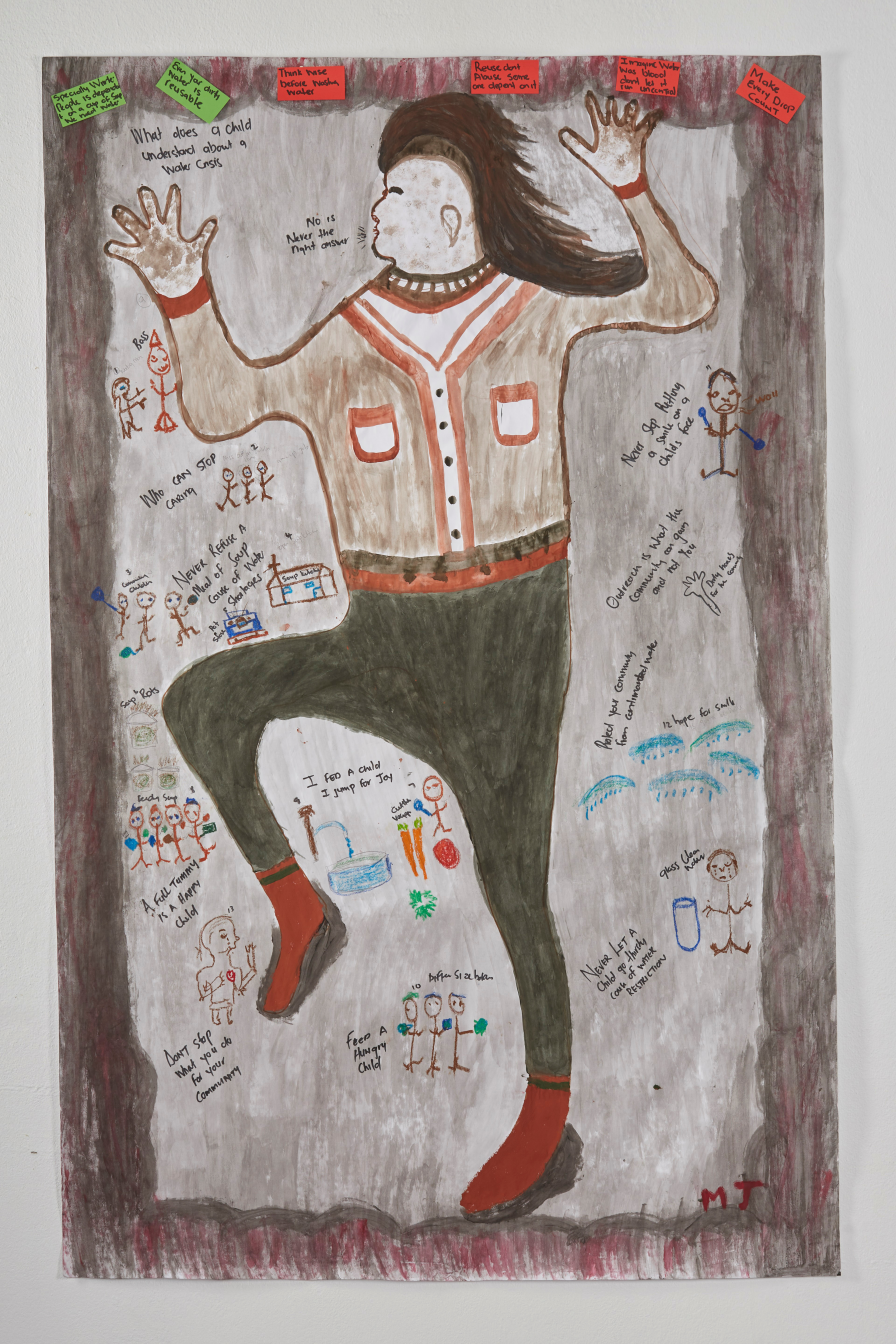
A body map describing the personal experiences and perceived implications of the 2018 Cape Town water crisis, produced by a community participant during the five-day creative workshop in Delft.

In the BLH body mapping process participants were asked to create life-size images reflecting their individual embodied experiences of water shortage (Figure 1). The body mapping approach that was followed has been described elsewhere (25). The creation of the body maps catalyzed the recollection of water scarcity, which became the building blocks for the narrative content of five collaborative films.

#### Sound

Music-making activities were woven throughout the five-day creative process. This included listening to water sounds and discussing responses, rhythm play with formal and improvised percussion instruments, singing and song-making. The music facilitator recorded all these activities and used the recordings to build new compositions, which were later used as soundtracks for the videos emerging from the workshops.

#### Roleplay

Both creative workshops also included a role-playing component, in which small groups developed short dramas about issues that had emerged regarding research and researchers. In Enkanini, these dramas focussed on community experiences of working with researchers in the settlement.

#### Video Production

The video-making phase of the creative workshops took different forms with the two focus groups. In Enkanini, filmed body map presentations evolved into a collaborative film, Our Water Challenges, about the problems of dirty water and waste, and the need for collective action.

In Delft, the filmed body map presentations became the basis of four short videos grouped around themes identified by the engagement team that reflected the diverse impacts of the water crisis: Children, Water and Recreation; Community Spirit; Health, Stress and Sanitation; and Water and Loss^2^.

#### Planning Workshops

Three planning workshops were held with each of the focus groups, during which they rehearsed the presentation of their visual materials and helped to design the engagement and exhibition events.

#### Research Team

**Figure 2:**
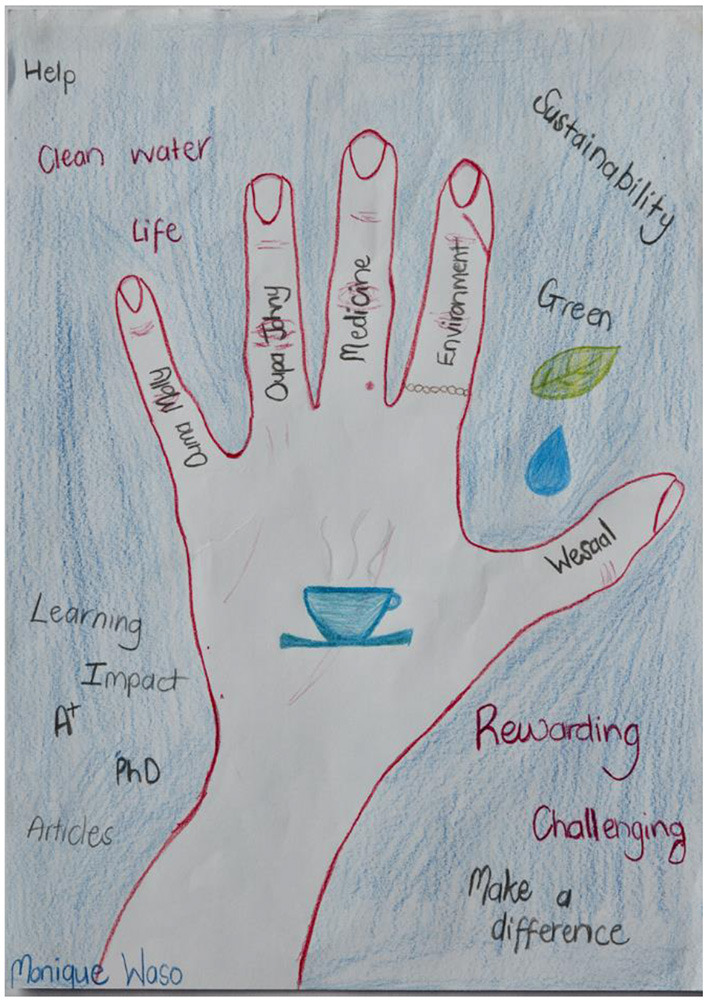
A hand map produced by a PhD student during a planning meeting with the engagement team at SLF; the fingers show five key factors influencing the student's decision to become a water microbiologist.

The engagement team led a one-day workshop at the SLF campus to help the microbiologists prepare for the knowledge exchange days and to develop research presentations that would be accessible to the community focus groups. The research team also created hand maps (26) as visual aids for introducing themselves (Figure 2). The engagement team had developed a hand mapping process for community safety research (27) that they adapted to fit the BLH project. Each scientist was asked to look at one of their hands and think of the fingers as representing the five major influences that had informed their decision to become a water microbiologist. More detail about the hand mapping method used in the BLH project is given in Appendix 1.

## Phase 2: Knowledge Exchange Days

Two knowledge exchange days were held in Stellenbosch, one with each community focus group.

**Figure 3:**
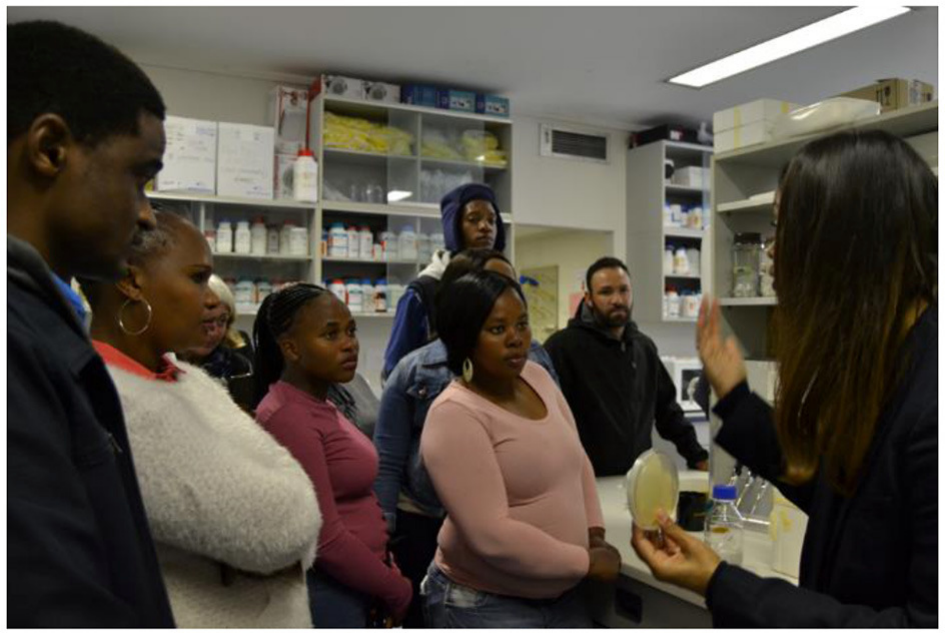
The head of the Water Resource Laboratory, Stellenbosch University, demonstrates the use of agar petri dishes to Enkanini participants as part of knowledge exchange activities.

The intention of the knowledge exchange days was to facilitate genuine two-way communication by creating situations in which all groups held roughly equal power and were required to listen and respond to each other. Researchers introduced themselves *via* their hand maps and delivered presentations covering the basics of water science and their own research. They also took each focus group on a guided tour of their research department and laboratory (Figure 3).

**Figure 4:**
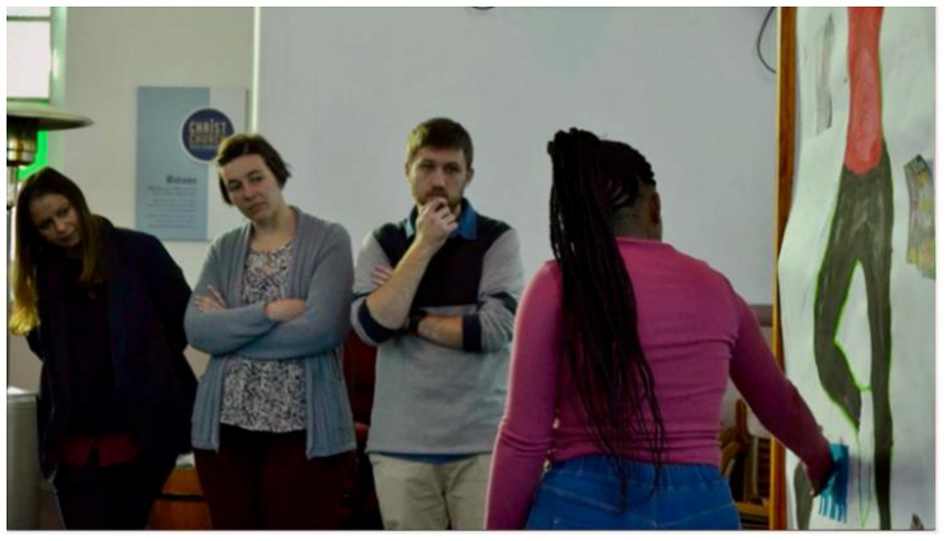
Researchers from the Water Resource Laboratory listen as an Enkanini community member presents her body map as part of knowledge exchange activities.

**Figure 5:**
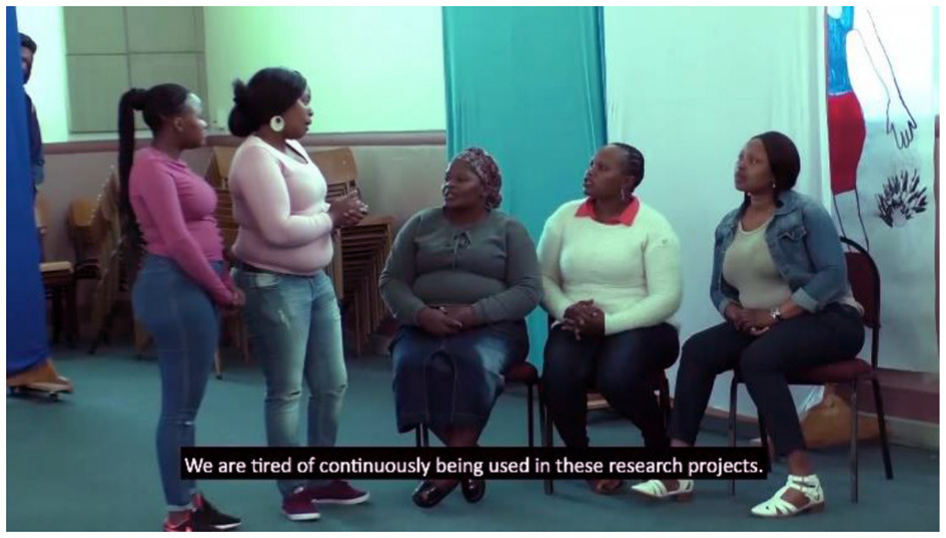
A clip from the film “Doing it Differently”, showing a moment in a role play performed by members of the Enkanini focus group for the Water Resource Laboratory team at the knowledge exchange event in Stellenbosch.

Each of the community members presented their own body map to the researchers (Figure 4). The Enkanini group also presented one of the short dramas they had developed during the creative workshop (Figure 5).

## Phase 3: Public Exhibitions

The final element of the engagement process comprised two public exhibitions, one at the public library in Delft and one at the HB Thom Theater in Stellenbosch. Both exhibitions showcased the community maps, body maps and films created by the focus group participants, as well as the microbiologists' and maps and scientific posters. Both exhibitions were attended by the scientific team and the focus group members, as well as other residents of the participating communities and researchers from multiple academic institutions.

The Enkanini focus group decided to use their collaborative film to raise consciousness about the disposal of wastewater and unused food in their community, and to inspire local behavior change. Our Water Challenges was shown to 20 Enkanini residents during a community mobilization event organized by the focus group and held in a small church hall in the settlement.

### Doing It Differently

To document the engagement process for sharing with other community engagement practitioners and researchers, SLF produced a 12 min film called doing it differently^3^. The film has been presented at several meetings and conferences around the world.

### Project Evaluations

The engagement team facilitated formative evaluation sessions with the community participants and microbiologists as part of the creative workshops. A summative evaluation was conducted with the Delft participants by an external evaluator from the Human Sciences Research Council at the end of the project.

## Discussion

BLH provided two central platforms for engagement between the community focus groups and the microbiology team: knowledge exchange days and public exhibition events. Although each of these events only lasted a single day, their contents were designed over many weeks. In this section, we discuss how effectively the core communication tools used at the engagement event fostered listening and catalyzed responsiveness. We then reflect on the most significant lessons learned through facilitating the BLH project.

### Body Maps

The body maps created by the community focus groups proved to be valuable materials for fostering storytelling and engagement. Creating these life-sized personal artworks instilled a sense of ownership among the participants and enabled them to convey their visceral experiences of water shortage, visually and verbally. In Enkanini, the body maps conveyed embodied experiences of exposure to dirty and contaminated water. In Delft, the body maps expressed various personal health and wellbeing challenges related to the 2018 water crisis. Participants presented their body maps and told their stories several times during the creative workshop process. These repeated presentations helped the community members to build their skill and confidence for telling their stories in public, and to reflect on and choose levels of self-disclosure. The body maps also made striking exhibits and proved to be effective conversation pieces during the knowledge exchange days and public exhibitions. To our knowledge, this was the first time that body mapping had been used as a tool for community engagement in water microbiology. At an evaluation session with the research team following the Enkanini knowledge exchange day, one of the PhD students noted:

“*I was always under the impression that the biggest struggle would be access to water, but looking at the body maps, the discussions and everything else today it became apparent that it's more an issue with the gray and black water and the health risks associated with that.”*

This researcher's response provides an example of how viewing material created through participatory visual methods can promote a more reflexive understanding of research practices' on the part of researchers (28).

### Hand Maps

Hand mapping with the scientific team helped to bring the researchers into the knowledge exchange days as equal participants, sharing life experiences and personal reflections through creative media. The resulting vulnerability was not always comfortable but did contribute to a leveling of power between the groups. In a workshop evaluation exercise, one of the microbiologists reported:

“*I finally understood that the presentation of our hand-maps “Humanized” us in a sense – we could reveal a bit of personal information and we were not just the scientific team!”*

### Drama Performance

The knowledge exchange day with the Enkanini focus group included a role-play session in which the group expressed their opinions about research being done in their community. Enkanini's proximity to the University of Stellenbosch makes it a frequent location for research projects across many different disciplines, which are not necessarily co-ordinated. The group expressed that occasional opportunities for a few residents to join projects as co-researchers were insufficient, and strongly expressed their expectation for compensation as research participants, as well as their wishes for further education and employment opportunities through the university. Thus, the community participants used the role play activity to express anger and frustration with the broader relationship between Enkanini and the university that they had not previously had a chance to vent. However, they did not do this as a personal attack upon the research group but rather to show their dissatisfaction with academic research in their community *per se*. After the performance, the engagement team facilitated a discussion that provided a space for the scientists to explain the scope and boundaries of their research programme, and how this prevented them from being able to provide the opportunities that were sought. Although the performance and subsequent discussion introduced some tension to the event, they did not undermine the process of two-way communication. On the contrary, these activities opened a difficult but needed conversation that generated important learning and understanding for the researchers, the Enkanini participants and the engagement team. The roleplay component reinforced the power of community-led drama to strengthen engagement in health science research (29).

### Collaborative Films

The five films that were co-created with the focus groups allowed them to work together and convey their collective experiences of water shortage in their communities. The distinctive video-making paths undertaken by the two groups reflected their differing circumstances, difficulties, priorities, and aspirations. The films proved to be effective tools for discussion and debate at all the events, underlining the advantages of participatory video approaches to engagement in health science research (30).

Our Water Challenges evoked a strong reaction and a call for action amongst the Enkanini residents who attended the community mobilization event. At this moment the project had the greatest potential to take on a life of its own. However, the focus group's attempt to expand their awareness-raising campaign was blocked by their own lack of resources, and the 12 months duration of BLH offered limited scope to support their initiative. This is a common frustration, reflecting the fact that communities function as ecosystems within which external financial and other resources are a critical source of the energy required to sustain any initiative for change (11).

### Participant Responses

Participants overall reported that they had enjoyed and benefited from the project. On a personal level, they felt validated and empowered by sharing their body maps and stories and indicated that they had gained important learning about water safety and water science. Strong interpersonal bonds were also formed among the Delft participants in particular. The desire to be included in decision making about research priorities was expressed by a focus group participant during the summative evaluation workshop:

“*I find it ironic that research is only done when an issue becomes critical but never done before or when new developments are in the pipeline. Things that matter to us are not researched in a manner that involves or engages us as community members.”*

In a reflection on the need for community engagement that involves listening to the experiences and perspectives of community members, participants expressed surprise and delight at the realization that they were able to teach the researchers something, as opposed to merely being the recipients of knowledge.

“*The experience was eye-opening as I got to experience the more academic side of the water cycle and what efforts scientists are doing, but it was also surprising to realize how much knowledge the students got from us.”*

The only major area of dissatisfaction expressed by the community members was a direct consequence of the relatively short duration and limited funding of the project. Members of both the Enkaninin and Delft focus groups expressed their aspirations to take their learning about water research from BLH further into their respective communities and lamented the lack of resources to allow this. Participants also conveyed regrets about the lack of scope to take their lived experiences of water shortage into interactions with other stakeholders.

An evaluation specialist who reviewed the project with the Delft participants noted:

“*The group felt that they still had a lot to learn, they wanted greater interaction with more researchers, and they wanted to engage with the public more, telling their stories and informing community members about what they had learned.”*

### Research Team Responses

The research team entered the project with high enthusiasm along with a degree of trepidation. As one member noted in an evaluation:

“*I was apprehensive at first particularly as I associated the project with social science. I do not always understand the reasoning or “Thought Process” of social scientists so I fully expected the interaction with the [engagement] team to be challenging. What did not help matters is that they wanted us to talk about our feelings during the hand-map session. My perception of the team changed when the first workshop [Knowledge Exchange Day] was presented to the Enkanini group and subsequently to the Delft group. This is when I became aware of how valuable the tools are that they employ.”*

The project enabled the microbiology team to see the significance of their research in a new light and to acknowledge the value and ethical obligation of in-depth community engagement. After the conclusion of the project, the microbiology team leader decided to withdraw WRL activities from Enkanini. This was an unforeseen response and an unintended consequence of the engagement process. The research team did not retreat because they were dissatisfied with the BLH project. They did so because BLH had revealed to them that residents of the informal settlement were aggrieved about the lack of community benefits arising from research in Enkanini (*per se*). Through the CEI project the microbiologists also recognized that alternative water sources may not be a high priority for Enkanini community members, whereas greywater treatment strategies were urgently required.

“*The high number of research projects being conducted in the settlement due to its proximity to the university made us realize that they [The Enkanini Project Participants] had every right to feel exploited*.”

Although the researchers seriously deliberated the possibility of including Delft as a new research site, because of the high rate of violent crime in the township the risks of doing fieldwork there were considered to be too high.

### Engagement Team Responses

The engagement team was committed to allowing the community participants and researchers to shape the activities and outputs of the engagement process. This required holding back on fully defining the project design in advance which, although an effective approach, inevitably introduced a level of uncertainty and unpredictability. Through collective experience, the engagement team was able to facilitate a reflexive and adaptable process. We would advise others embarking on similarly open-ended projects to expect similar uncertainty and to build and capacitate their engagement teams accordingly. On reflection, and with regards to the two-way communication aspect of CEI, we would argue that core standards should include the possibility for feedback from all participants to alter the pathway of engagement. In addition, we regard that to be considered successful, CEI should result in the exchange of information that promotes new learning, is acknowledged as valuable and is actionable by those involved in the exchange process.

In its self-evaluation, the engagement team also noted its own positionality and limited diversity. The only person of color in the engagement team withdrew in the early stages of the project due to circumstances beyond his control and there were no fluent isiXhosa speakers in the team. The continual presence of a isiXhosa-speaking observer during the Enkanini workshops was valuable, as was the involvement of participants who were acknowledged community leaders or had previous experience as community-based researchers, which helped to mitigate disparities of power.

### Constraints of the Study

From our perspective, a main methodological constraint of the project was linked to its time frame. There was a substantial imbalance between the time and effort required to generate the communication tools, especially those produced by the two community focus groups, and the actual interaction between the focus groups and the research team. Whilst the knowledge exchange days offered effective interfaces for mutual listening and co-learning, the one-day timeframe of these crucial events limited possibilities for reflection and whole-group discussions about appropriate and pragmatic responses to the learning gained by all participants.

The short duration of the project exhibition events also curbed the level of interaction between the participants and external stakeholders. Although these two forums provided a further opportunity for two-way communication between the research team and community residents, this opportunity was diluted by the presence of others.

We conclude that research institutions, as well as sponsors and funders (2) need to substantiate their professed commitments to community engagement by planning and budgeting for adequate time, support and resources to make such engagement meaningful. Neglect to do so risks the integrity of CEI and could foster a perception that community engagement is “Window Dressing”.

The political nature of access to safe water and sanitation was a key conceptual driver for the project, and a core objective was to bring the everyday impacts of water shortage in the participating communities, and the research of the Water Resource Lab, to the attention of local government. The engagement team tried multiple times to invite influential representatives from the Department of Water and Sanitation (DWS) to the exhibition event in Delft. The department did send a junior officer who arrived after the community members had presented their body maps. The officer delivered a promotion of the municipal water-saving campaign which was inappropriate following the emotional presentations given by the focus group. Although taken to task by members of the audience, the government representative did not have the power to escalate the group's complaints about the challenges of enforced water restrictions to decision-makers in his department. The project was purposively carried out during the 2018 Western Cape water crisis and at that time, the CoCT DWS was under extreme pressure to respond to unprecedented drought conditions. It is likely that senior representatives of this department were therefore unavailable to attend events that did not directly address the crisis situation. Whereas, SLF did not have an established link with the DWS, the Foundation was connected with several other CoCT departments. Although it may have been possible to better draw upon these existing connections to link to senior officials in the DWS and organize meetings beyond the exhibition event (31) the circumstances at the time would probably have made this particularly difficult. This scenario provides an example of the challenges that community engagement projects face in being able to influence government responsiveness (32) especially when engagement is addressing a current emergency situation.

### Surfacing Vulnerabilities

It has previously been shown that using participatory visual methods to explore connections with water and water governance can surface emotional responses (33, 34). The far-reaching consequences of water shortage surfaced deep and sometimes unanticipated vulnerabilities amongst the BLH community participants. Within the Delft focus group, the body mapping process revealed that a participant had witnessed the deaths of several family members in a house fire due to the lack of water to fight the blaze. Other participants offered strong support which enabled the activities to continue, and the engagement team arranged counseling as per SLF organizational policy. This outcome highlighted the ethical complexity of PVM practice in health science engagement (25, 35) and the need for levels of responsiveness that go beyond the conceptual boundaries of engaged research.

The project also confronted the research team members with their incapacity to make a difference in immediate and tangible ways. In most instances, research groups do not have the scope, training, or funding to be responsive to local populations' needs, conditions, and concerns, especially when these are largely structural in nature. This powerlessness can leave researchers feeling conflicted, ethically concerned, and unable to do what they feel is right (36) and may have detrimental effects on researcher motivation. Appropriate support structures such as debriefing and ethics discussion groups (36) for research teams and community members alike could substantively strengthen the outcome of community engagement initiatives. We would also encourage research groups to consider that moments of conflict and discomfort present opportunities for more meaningful engagement and should not be avoided or suppressed.

## Conclusion

The BLH project has shown how the participatory creation of personal and collective visual materials, by both researchers and community members, can foster effective CEI and increase engagement integrity. Materials such as community maps, body maps, hand maps and tailored scientific presentations provided effective platforms for reciprocal listening and co-learning. Roleplay performance by community members helped to surface important points of tension and stimulate valuable and needed conversations. Visits to the Water Resource Laboratory were appreciated and enjoyed by the Enkanini and Delft participant groups, and further strengthened interaction and learning. The entire engagement process catalyzed verbal and active responses by the researchers and focus group members. However, further outcomes desired by participants were constrained for multiple reasons, including the limited timeframe and resources of the project and factors that were beyond the control of the community participants, the microbiologists and the engagement team. Policy engagement was almost entirely obstructed by a lack of acknowledgment from local government.

The project has shown that building engagement integrity through participatory visual methods requires a substantial initial investment of time and preparation, especially when engaging with marginalized communities. This can detract from the time available for knowledge exchange and public engagement. The case study also highlights the need for CEI to be reflexive and open to going in unforeseen directions.

The core standards recommended for CEI by global organizations (3, 4) carry no real weight unless participation, empowerment and ownership, inclusion, two-way communication, adaptability and localization, and capacity development are realized to the satisfaction of the communities where research is undertaken. Are funders, researchers, engagement practitioners, community members and government bodies ready to work in partnership and commit the time, energy, resources, and humility that will be required to achieve true engagement integrity?

## Data Availability Statement

The raw data supporting the conclusions of this article will be made available by the authors, without undue reservation.

## Ethics Statement

Ethical review and approval was not required for the study on human participants in accordance with the Local Legislation and Institutional Requirements. The patients/participants provided their written informed consent to participate in this study. Written informed consent was obtained from the individual(s) for the publication of any potentially identifiable images or data included in this article.

## Author Contributions

The community case study was conceived and led by GB and PS contributed to the study design and facilitated the participatory workshops and engagement events. GB wrote the first draft of the manuscript. PS wrote sections of the manuscript. Both authors contributed to manuscript revisions, read, and approved the submitted version.

## Funding

This community case study was supported by the Wellcome Public Engagement Fund (Grant No. 209586_Z_17_Z).

## Conflict of Interest

The authors declare that the research was conducted in the absence of any commercial or financial relationships that could be construed as a potential conflict of interest.

## Publisher's Note

All claims expressed in this article are solely those of the authors and do not necessarily represent those of their affiliated organizations, or those of the publisher, the editors and the reviewers. Any product that may be evaluated in this article, or claim that may be made by its manufacturer, is not guaranteed or endorsed by the publisher.

## Footnotes

^1^SLF is a Non-Governmental Research Organization based in Cape Town, South Africa https://livelihoods.org.za/.

^2^The files for the four themed videos produced with the delft participants are too large to upload as Supplementary Material. They can be viewed on YouTube using the following links: Children, Water and Recreation https://www.youtube.com/watch?v=Wk9B2FhqHFM, Community Spirit https://www.youtube.com/watch?v=vDbmr-KKSIM, Health, Stress and Sanitation https://www.youtube.com/watch?v=5pyhtAoNb38, Water and Loss https://www.youtube.com/watch?v=5pyhtAoNb38&t=76s.

^3^The video file for Doing it Differently is too large to upload as Supplementary Material. The video can be viewed on YouTube at Doing it Differently https://www.youtube.com/watch?v=-_7jAhOgGGU&t=3s.
