# Supplementary material for: Steps Toward Engagement Integrity: Learning From Participatory Visual Methods in Marginalized South African Communities
Source: Front Public Health. 2022 Jun 27;10:794905. doi: 10.3389/fpubh.2022.794905 (PMC9272585; doi:10.3389/fpubh.2022.794905)
Supplement: Supplementary file 1 [file Data_Sheet_1.pdf]

Appendix 1: Steps towards engagement integrity: learning from participatory visual methods in marginalized South African communities

Process and guiding questions for the creation of hand maps for the Bucket Loads of Health project by members of the Water Resource Laboratory at Stellenbosch University

Introduction

Our hands silently say so much about us. Although we use them nearly all day every day for doing something or other, we don't really pay that much attention to what we are doing with them. But what we do with our hands at work, at home and for recreational purposes, defines us more than any other part of our body, apart from perhaps our minds. Thinking about our hands then, is a great way to start thinking about ourselves, and for entering into a process of self-reflection.

Take some time to look at your hands. Look at them slowly and carefully, on both sides. Think about the many different things that you do with them.

Maybe you are wearing a ring? Or perhaps you have scratches, or other marks that remind you about an experience you have had at some point in the past.

Method :

Using a dark coloured pen or pencil, draw one of your hands on an A4 piece of white paper (trace the outline or represent your hand with a free-style drawing).

Add in any scars, marks, rings, wrinkles, lines or other things that you are wearing - or that show on your hands. You might want to sketch in your nails, and perhaps your knuckles.

Choose a colour (or multiple colours) for the background around the outside of your hand and fill it in.

Write your first name clearly in one corner of your hand map.

Look at the inside of you hand and pay attention to the way that all your fingers come together and intersect at the palm of your hand.

Imagine yourself as a mini-you, sitting there in the palm of your own hand. In the centre of your hand on your hand map, draw a symbol, a picture, a letter, or something else that in some way symbolizes you.

Think of your fingers as representing the top five major influences in your life that steered your decision to become a water microbiologist. Here we are talking about history; things from the past that have driven you on the path to becoming a scientist, and in particular a water microbiologist. It can be anything at all that has been significant and important to you.

Take as much time as you want to reflect on this, and then - inside the fingers of your hand map, and using a dark coloured pen or pencil - write (or draw) each one of these five major influences in one or two words.

Think about what drives you forward as a scientist. What contribution do you want to make to the world as a consequence of the work that you do? What do you want to achieve? What does success

look like to you? Illustrate these contributions, achievements or successes in words, pictures, symbols - or however you want to - in different places in the background of your hand map.

Write a short paragraph of text (it can be in English, Afrikaans or isiXhosa) that describes these contributions, achievements or successes. This text will accompany your hand map when you present it to the community participants at the Knowledge Exchange workshops, or when it is shown at project exhibitions and events.

Your hand map will not be presented or shown without your informed consent.
